# Glycoprotein non-metastatic melanoma protein B is a potential biomarker for arthroplasty aseptic loosening

**DOI:** 10.1038/s41598-025-13922-3

**Published:** 2025-09-12

**Authors:** Patrik Schadzek, Alexander Derksen, Wiebke Behrens, Maike Kosanke, Oliver Dittrich-Breiholz, Anika Hamm, Kirsten Elger, Yvonne Roger, Ines Yang, Meike Stiesch, Yvonne Noll, Marco Haertlé, Lars-René Tuecking, Christina Stukenborg-Colsman, Henning Windhagen, Doan Duy Hai Tran, Anette Melk, Andrea Hoffmann

**Affiliations:** 1 Department of Orthopaedic Surgery, Biological Basics for Biohybrid Implants OE 8893, Hannover Medical School, Anna-von-Borries-Str. 1-7, Stadtfelddamm 34, 30625 Hannover, Germany; 2 Department of Orthopaedic Surgery, Hannover Medical School and DIAKOVERE Annastift, Anna-von-Borries-Str. 1-7, 30625 Hannover, Germany; 3 Department of Prosthetic Dentistry and Biomedical Materials Science, Hannover Medical School, Carl-Neuberg-Str. 1, 30625 Hannover, Germany; 4 Research Core Unit Genomics, Hannover Medical School, Carl-Neuberg-Str. 1, 30625 Hannover, Germany; 5 Department of Nephrology and Rheumatology, University Medical Centre Göttingen, Robert-Koch-Str. 40, 37075 Göttingen, Germany; 6 Department of Paediatric Kidney, Liver and Metabolic Diseases, Hannover Medical School, Carl-Neuberg-Str. 1, 30625 Hannover, Germany; 7 Lower Saxony Centre for Biomedical Engineering, Implant Research and Development (NIFE), Stadtfelddamm 34, 30625 Hannover, Germany; 8 DZHK German Centre for Cardiovascular Research, Partner Site Lower Saxony, Göttingen, Germany

**Keywords:** Biomarker, Defect length, Endoprosthesis loosening, Extracellular domain shedding, Osteoactivin, Transcriptome analysis, Molecular biology, Biomarkers, Diseases, Health care, Medical research

## Abstract

Endoprosthesis loosening is the major cause of arthroplasty failure. Currently, radiography, histo-pathological classification of the periprosthetic membrane, and microbiological examination are used retrospectively for diagnosis. Prospective options for early diagnosis are not available. The study presented here aimed to identify (prospective) molecular biomarkers of implant loosening in tissue samples from patients. Four patient cohorts (primary and revision arthroplasty due to aseptic loosening of hip or knee) were defined. Aseptic loosening of implants was assessed by the standardised approach of the Knee Society Total Knee Arthroplasty Roentgenographic Evaluation and Scoring System. Synovial fluid, bone marrow, and blood were collected from 96 patients. Mesenchymal stromal cells (BM-MSCs) were isolated from the bone marrow. Bulk RNA-sequencing of 28 samples (including a fifth patient cohort with knee arthrofibrosis as aseptic cause for revision surgery) showed that Glycoprotein Non-Metastatic Melanoma Protein B mRNA (*GPNMB*) was significantly upregulated in BM-MSCs derived from revision patients compared to patients with primary implantations. Elevated *GPNMB* mRNA levels were confirmed with qRT-PCR. Synovial fluid plasma study by ELISA revealed increased levels of GPNMB protein in patients undergoing revision surgery compared to patients undergoing primary arthroplasty. GPNMB concentration in synovial fluid plasma, which can be obtained non-invasively, could be a potential biomarker for early detection of implant loosening, ahead of current diagnostic procedures.

**Supplementary Information:**

The online version contains supplementary material available at 10.1038/s41598-025-13922-3.

## Introduction

Hip and knee endoprostheses (THA, TKA) are major implant types in orthopaedic and trauma care. In 2022, 177,826 hip and 137,030 knee primary surgeries were performed in Germany, and additionally 18,145 hip/14,379 knee revision surgeries^1^.

Loosening and the resulting implant failures of endoprostheses pose a significant challenge^2^. For the diagnosis of looseness, either a skeletal scintigraphy or radiological images in at least two planes are performed^3,4^. In this context, radiolucent lines in the latter can indicate loosening^2^. If these progress over time, this indicates a nearly certain sign of implant failure^2^. With the help of radiographic evaluation systems, particularly the Knee Society Radiographic Evaluation System (KSRESS), signs of loosening in knee prostheses are assessed uniformly^4,5^. Aseptic loosening is responsible for 55% of cases, septic loosening due to infections accounts for about 15%, and other reasons like periprosthetic fractures or implant dislocations make up the remaining 30% ^1,6^. Due to the high proportion of aseptic loosening, the current study focuses on this pathology.

Aseptic loosening is characterised by osteolysis due to increased osteoclast activity, resulting in insufficient osseointegration of the endoprostheses. Moreover, the process includes a reparative and inflammatory response of the immune system^7^finally resulting in fibrosis. This is histo-pathologically classified as type IV implant failure according to Krenn and Morawietz^8^. A major trigger seems to come from wear particles released during motion of the joint, histo-pathologically classified as type I implant failure. Type I and type IV failure occur in combination in most cases of joint arthroplasty aseptic loosening.

Early detection of implant loosening is currently not possible. Thus, there is an urgent need for novel diagnostic parameters including individual biomarkers or complex signatures. Following this rationale, the study presented here focused on the unbiased transcriptome analysis of mesenchymal stromal cells from bone marrow (BM-MSCs) as one prominent type of regenerative cells for implant-tissue interaction. BM-MSCs of patients undergoing primary or revision surgery for hip or knee endoprostheses were analysed (in total 28 samples). Subsequent confirmation of potential biomarker candidates was performed in body fluid plasma on protein level to pinpoint protein biomarker candidates for revision arthroplasties, based on material from 96 patients.

## Materials and methods

### Patient cohorts

#### Clinical information

This study included 96 patients who received primary hip or knee arthroplasty due to osteoarthritis, or revision hip or knee surgeries due to aseptic implant loosening as diagnosed by roentgenographic assessment (cf. below) between 2021 and 2024 in the study hospital. Exclusion criteria were infections with hepatitis viruses or MRSA, depressions, strong smoking, and tumour diagnoses. Intraoperatively, synovial fluid, bone marrow, and blood were targeted in all patients but did not result in successful procuration in all cases. Condensed patient information is displayed in Table 1 (for more details, please refer to Suppl. Table 1). Five cohorts were defined from a subset of the study group for RNA-sequencing of BM-MSCs: primary hip (PH, 4 cases for the pilot run, 12 cases in total, colour code: light grey), revision hip (RH, 2 cases for the pilot run, 2 cases in total, colour code: dark grey), primary knee (PK, 2 cases for the second run, colour code: blue), revision knee (RK, 2 cases for the pilot run, 7 cases in total, colour code: violet), and revision knee due to arthrofibrosis (RK AF, 5 cases for the second run, colour code: pink). The four major cohorts (arthrofibrosis exempted) with inclusion of more patients were used for biochemical assays (ELISA, total protein).

The BMI of the cohort with primary hip implantations was 28.0 ± 3.9 (range 22.1–34.9) for 23 patients (7 male, 16 female). The BMI of the cohort with primary knee implantations was 31.0 ± 5.2 (range 23.4–39.3) for 20 patients (10 male, 10 female). The BMI of the cohort with revision hip implantations was 27.2 ± 6.8 (20.0-51.9) for 25 patients (13 male, 12 female). The BMI of the two cohorts with revision knee implantations (arthrofibrosis included) was 31.8 ± 6.6 (21.3–54.9) for 28 patients (12 male, 16 female). In summary, the body mass index of patients with knee surgeries was higher than with hip surgeries.

**Table 1:** Patient information.

	Primary Hip	Revision Hip	Primary Knee	Revision Knee	Arthrofibrosis
	(*n* = 23)	(*n* = 25)	(*n* = 20)	(*n* = 23)	(*n* = 5)
Age (years)					
Mean	69.7	67.2	68.7	67.7	65.6
Standard deviation	9.6	15.3	11.4	12.0	8.6
Range	55–86	22–83	41–88	41–90	60–80
Sex					
Female, No. (%)	16 (70)	12 (48)	10 (50)	13 (57)	3 (60)
Male, No. (%)	7 (30)	13 (52)	10 (50)	10 (43)	2 (40)
BMI (kg/m²)					
Mean	28.0	27.2	31.0	32.1	30.3
Standard deviation	3.9	6.8	5.2	7.0	4.5
Range	22.1–34.9	20.0–51.9	23.4–39.3	21.3–54.9	23.8–35.4

BMI: body mass index.

**Fig. 1:**
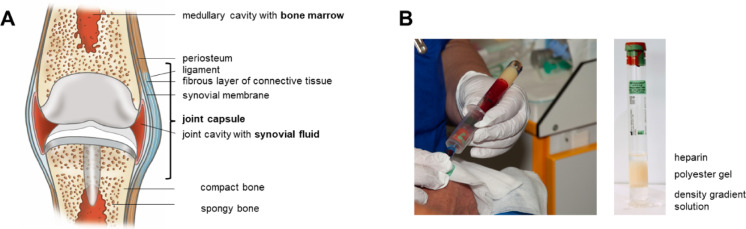
Tissue sampling. (**A**) Sagittal section through hypothetical synovial joint with hypothetical implant. (**B**) Blood sampling was optimised using an adapter (left) combined with a CPT™ tube (right) for density gradient preparation of plasma and MNCs.

Informed consent was received prior to participation. For the photo in Fig. 1B, written informed consent was received for the use of the photographs and publication. All research was performed in accordance with relevant guidelines/regulations.

#### Sex as a biological variable

Our study examined male and female patients. No significant differences in overall expression patterns were observed from RNA sequencing data in comparisons of all 28 female and male patients (PERMANOVA, *p* > 0.05), both with and without control for other technical and biological variables. Nevertheless, tested groups were either balanced for sexes, or sex was included as potential co-variate in RNA-sequencing analyses when testing primary and revision arthroplasty samples of hip or knee for overall differences in expression patterns (PERMANOVA) and differentially expressed genes (DESeq2).

#### Age as a biological variable

All 28 patients whose BM-MSCs were used for RNA-sequencing were aged 60 years or above, with the exception of one younger person. The 87 patients whose body fluids were assessed by ELISA were aged 54 years or above, with the exception of 6 younger donors. No significant differences in overall expression patterns of RNA-sequencing data were observed in relation to age (PERMANOVA, *p* > 0.05). Tested groups were either balanced for age, or age was included as potential co-variate in analyses of overall differences in expression patterns (PERMANOVA) and of differentially expressed genes (DESeq2) when testing for differences in primary and revision arthroplasty samples of hip or knee.

#### Roentgenographic assessment of implant loosening

Characteristic structural changes in osteoarthritis, their severity and their accompanying phenomena can be easily detected by X-rays, as radiolucent lines at the boundary between the prosthesis and the neighbouring bone or bone cement interface, as well as between the bone cement and the bone^9^. The radiolucent lines were assessed using the standardised approach of the KSRESS. The assessment was conducted with standardised X-ray pictures in the anteroposterior and axial positions of the knee (cf. Suppl. Fig. S1). All spots that were transparent to X-rays and had a diameter larger than 1 mm were noted and documented based on their corresponding zones^9^. Consequently, the overall defect length was determined by adding the measurements of each individual zone. In the anteroposterior pictures, three additional zones were included for cases with existing stems. This seems rational as evaluating tibial stems necessitates an assessment in imaging planes that are oriented differently^10^. In order to mitigate distortions caused by magnification effects, the OrthoView program (Jacksonville, FL, USA) was employed to examine and standardise the true magnification of the X-ray pictures.

The radiological assessments were carried out separately by three skilled orthopaedic surgeons. A study on intra-observer reliability was conducted, yielding an intraclass correlation coefficient of 0.83, indicating a significant degree of measurement reliability.

#### Biomaterial processing

An anatomical sketch of a hypothetical synovial joint with an implant is shown in Fig. 1A. Bone marrow, synovial fluid and blood were collected into heparin-containing tubes in order to prepare plasma and mononuclear cells (MNCs) from identical material as described in detail below.

### Bone marrow

Bone marrow was extruded from the femur and collected into syringes with heparin (2,500 IU/mL heparin per 10 mL of bone marrow). The bone marrow preparations were further diluted fourfold with phosphate-buffered saline without Ca^2+^, Mg^2+^ (PBS) and processed as described^11^. Bone marrow plasma was collected, thoroughly mixed, and then stored at − 80 °C in three aliquots until analysis. All plasma samples were slightly haemolytic.

The MNCs floating on the density gradient were processed into BM-MSCs as described^11^. For RNA-Seq, BM-MSCs were used in passage 3.

### Synovial fluid and blood

Synovial fluid and blood (Fig. 1B) were collected in BD Vacutainer^®^ CPT™ mononuclear Cell Preparation Tubes with an adapter. These tubes contain heparin and a density gradient material, hereafter called CPT™ tubes. The use of an adapter helped to minimise haemolysis and thereby notably improved the sample quality. After centrifugation at 1700 x g for 25 min the plasma was recovered and stored at − 80 °C until analysis. The overall degree of haemolysis of synovial fluid from TKA patients was lower than from THA patients. In case of low volumes of synovial fluid, PBS was added into the CPT™ tubes before further processing in the laboratory since the tubes are designed for use with volumes of 8 ml.

The MNCs obtained during density gradient centrifugation were resuspended in residual plasma followed by centrifugation at 300 × g for 5 min at room temperature with brakes. The cell pellets were resuspended in freezing medium (95% FCS, 5% DMSO) and stored at − 140 °C in aliquots of 1 mL. For RNA isolation, aliquots were thawed in 9 mL ice-cold macrophage growth medium (M0-GM: RPMI-1640 (Sigma #R0883) with 10% FCS, 2 mM L-glutamine (Sigma G7513), 17.1 mM HEPES, 1% MEM non-essential amino acids (Sigma #M7145), 85.65 µM 2-mercaptoethanol, 100,000 U/L penicillin, 100 mg/L streptomycin, 25 ng/mL recombinant human M-CSF (Miltenyi Biotech #130-096-492, from *E. coli*)). Cells were incubated on ice for 10 min, then centrifuged at 300 x g for 5 min. The pellets were resuspended in 3 ml M0-GM, transferred into one well each of a 6-well plate and grown to about 90% of cell density until lysis for RNA isolation.

### RNA isolation, quantification and quality control

RNA was extracted from cell cultures via Trizol reagent (Invitrogen: 1 ml per 25 cm^2^ culture surface) according to the instructions by the manufacturer. Concentration was measured with a NanoDrop ND-1000 instrument. Samples for RNA-sequencing were subjected to a quality control with Agilent 2100 Bionanalyzer, using the Agilent RNA 6000 Pico Assay. Only samples with RNA integrity number > 7 were processed for RNA-sequencing.

### RNA-sequencing: library generation, quality control, and quantification

300 ng of total RNA per sample were utilized as input for mRNA enrichment procedure with ‘NEBNext^®^ Poly(A) mRNA Magnetic Isolation Module’ (E7490L; New England Biolabs) followed by stranded cDNA library generation using ‘NEBNext^®^ Ultra II Directional RNA Library Prep Kit for Illumina’ (E7760L; New England Biolabs). All steps were performed as recommended in user manualE7760 (Version 1.0_02-2017; NEB) except that all reactions were downscaled to 2/3 of initial volumes.

cDNA libraries were barcoded by dual indexing approach, using ‘NEBNext Multiplex Oligos for Illumina – 96 Unique Dual Index Primer Pairs’ (6440S; New England Biolabs). All generated cDNA libraries were amplified with 8 of final pcr.

One additional purification step was introduced at the end of the standard procedure, using 1.2x ‘Agencourt^®^ AMPure^®^ XP Beads’ (#A63881; Beckman Coulter, Inc.). Fragment length distribution of individual libraries was monitored using ‘Bioanalyzer High Sensitivity DNA Assay’ (5067 − 4626; Agilent Technologies). The quantification of libraries was performed by use of the ‘Qubit^®^ dsDNA HS Assay Kit’ (Q32854; ThermoFisher Scientific).

### Library denaturation and sequencing run

Equal molar amounts of individually barcoded libraries were pooled for, in total, 2 sequencing runs, in which each analyzed library constituted around 7.8% of overall flowcell / run capacity. The library pool was denatured with NaOH and was finally diluted to 1.8 pM according to the Denature and Dilute Libraries Guide (Document # 15048776 v02; Illumina). 1.3 mL of the denatured pool was loaded on an Illumina NextSeq 550 sequencer using a High Output Flowcell (400 M cluster) for single reads (20024906; Illumina). Sequencing was performed with the following settings: Sequence reads 1 and 2 with 38 bases each; Index reads 1 and 2 with 8 bases each.

### BCL to FASTQ conversion

BCL files were converted to FASTQ files using bcl2fastq Conversion Software version v2.20.0.422 (Illumina).

### Raw data processing and quality control

Raw data processing was conducted by use of nfcore/rnaseq (version 1.4.2), which is a bioinformatics best-practice analysis pipeline used for RNA sequencing data at the National Genomics Infrastructure at SciLifeLab Stockholm, Sweden. The pipeline uses Nextflow, a bioinformatics workflow tool. It pre-processes raw data from FastQ inputs, aligns the reads and performs extensive quality-control on the results. The genome reference and annotation data were taken from GENCODE.org (Homo sapiens: GRCh38.p13; release 34).

### Analyses of gene expression patterns based on RNA-sequencing data

Individual genes were tested for differential abundances in different study groups with DESeq2 using the Wald test (version 1.32.0)^12^. The DESeq2 design formula contained the sequencing run as possible technical co-variate (set of 28 samples was distributed over two independent sequencing runs), and sex and age in years as non-technical co-variates to control for unwanted variation for the large cohort of 28 samples while this adjustment was not necessary for the balanced pilot discovery cohort of 8 samples. Analysing the large cohort of 28 samples, all genes with at least 10 reads in two samples were included in the DESeq2 analyses.

For heatmap visualisation the software Qlucore Omics Explorer version 3.9 (Qlucore, Lund, Sweden) was used. The genes in the heatmap were sorted by their statistics (Welch´s t-test comparison between primary and revision cohorts).

Gene set enrichment analysis was performed with the ShinyGO 0.80 graphical gene-set enrichment tool^13^with differentially expressed genes from DESeq2 analysis as input, including all genes with significant different counts indicated by adjusted *p* values < 0.05 between primary and revision samples. ShinyGO 0.80 includes Kyoto Encyclopaedia of Genes and Genomes (KEGG: https://www.kegg.jp/;^14^ and Gene Ontology datasets.

### Quantitative real-time PCR analysis for selected genes

200 ng of the RNA was digested with DNase I and converted into cDNA by standard procedures. qRT-PCR was performed using the following gene assays purchased from Life Technologies: RPS29 Hs03004310_g1 (housekeeping gene), GPNMB Hs01095669_m1, and SDC4 Hs01120908_m1.

### ELISA for GPNMB and SDC4

The plasma samples from the different body fluids were thawed, briefly vortexed and centrifuged at 10,000 x g for 10 min at room temperature in order to remove debris. Afterwards, sandwich ELISA was performed to analyse the secretion of GPNMB (Human Osteoactivin/GPNMB DuoSet ELISA; DY2550, R&D Systems) or SDC4 (Human Syndecan-4 DuoSet ELISA; DY2918, R&D Systems) according to the manufacturer’s protocols.

Three aliquots of bone marrow plasma, duplicate aliquots of blood plasma, and one aliquot of synovial fluid plasma were assessed in duplicates. A close inspection of the bone marrow samples revealed small, solid insoluble particles, which rapidly sedimented after mixing. In order to remove these particles, all plasma samples were again centrifuged at 200 x g for 5 min, the supernatants were transferred into fresh tubes, immediately diluted 1: 10 and processed for ELISA. Blood plasma was diluted 1: 50 for GPNMB ELISA. Synovial fluid plasma was diluted 1: 3, 1: 10, or 2: 250 for GPNMB ELISA, as applicable to match the valid concentration range supplied by the standard concentrations. One sample was measured undiluted. For SDC4 ELISA, synovial fluid was diluted 1: 2 or used undiluted, bone marrow was diluted 1: 3 or used undiluted. ELISAs were evaluated with a four-parameter logistic fit by the open source tool myassays.com.

Synovial fluid was retrieved from two patients for reasons other than knee surgery. Four volumes were mixed with one volume of blood obtained from the same patients on the same day. These samples were used unprocessed or, after centrifugation, the respective plasma specimens for GPNMB ELISA in order to check for blood-dependent interferences with ELISA performance.

The total protein content (cf. below) of all samples was determined as a reference. The mean and standard deviation are reported in the figures.

### Total protein determination

Protein quantification was performed according to an adapted Bradford assay^15^ using bovine serum albumin as standard, directly after thawing the plasma samples. Total protein determination and final readout of relative GPNMB levels were important particularly for the synovial fluid samples due to dilution with non-defined volumes of PBS, which was required to match the volume capacity of the CPT™ tubes.

### SDS PAGE analysis and Western blotting

2 µg total protein per lane was applied per plasma sample onto 10%T polyacrylamide gels in reducing sample buffer. Per cohort of synovial fluid plasma and blood plasma, four samples were randomly chosen for analysis. For bone marrow plasma, all available samples were studied. After blotting to 0.45 μm polyvinyldifluoridone membranes, a total protein stain to confirm equal loading was performed with Ponceau S. As positive controls, the recombinant ELISA standard protein (a highly purified NS0-expressed recombinant human Osteoactivin/Fc Chimera fusion protein from the Human Osteoactivin/GPNMB DuoSet ELISA, molecular weight about 115,000–125,000 g/mol), a commercially available *E. coli*-expressed recombinant GPNMB (amino acids 22–474 with N-terminal His-tag, molecular weight 53,200 g/mol) or bone marrow plasma for endogenous GPNMB were used. Upon reducing SDS PAGE analysis, the recombinant Fc chimera has an expected molecular weight of 115,000–125,000 g/mol (information on chimeras by the manufacturers). Full-length variants of endogenous human GPNMB have been observed with 115,000 and 90,000 g/mol^16^. A C-terminal fragment is about 20,000 g/mol^17^. For detection of GPNMB, two different antibodies were used. One antibody was identical to the ELISA capture antibody (goat anti GPNMB, R&D Systems #AF2550). The second antibody was mouse anti GPNMB (LSBio/Biozol #LS-B6406). The blots were treated with secondary horseradish peroxidase-conjugated antibodies. Detection of horseradish peroxidase activity was performed with Radiance Q substrate (azure biosystems) in the Azure c600 imaging system unit (azure biosystems).

### Statistical analysis

Statistical analysis of sex and age as biological variables by PERMANOVA analysis is explained with the patient cohorts. For the statistical comparisons between two groups (primary vs. revision) the parametric unpaired Welch’s t-test was used. Experimental data are shown as individual values. Results were considered statistically significant if *p* < 0.05. GraphPad Prism 9.5.1 for Windows (GraphPad Software, Boston, MA, USA) was used for statistical analyses. The Spearman correlation analysis was also used for the relative GPNMB levels in synovial fluid and the normalized counts of the GPNMB expression in BM-MSCs. This coefficient does not require a normal distribution of variables and can also measure non-linear monotonic relationships. For the correlation analyses between BMI and relative GPNMB levels in synovial fluid, the Pearson correlation was used. The data were tested for normal distribution using the Shapiro-Wilk test.

## Results

### BM-MSCs demonstrate notable gene expression differences between primary and revision surgeries including genes encoding soluble proteins

**Fig. 2:**
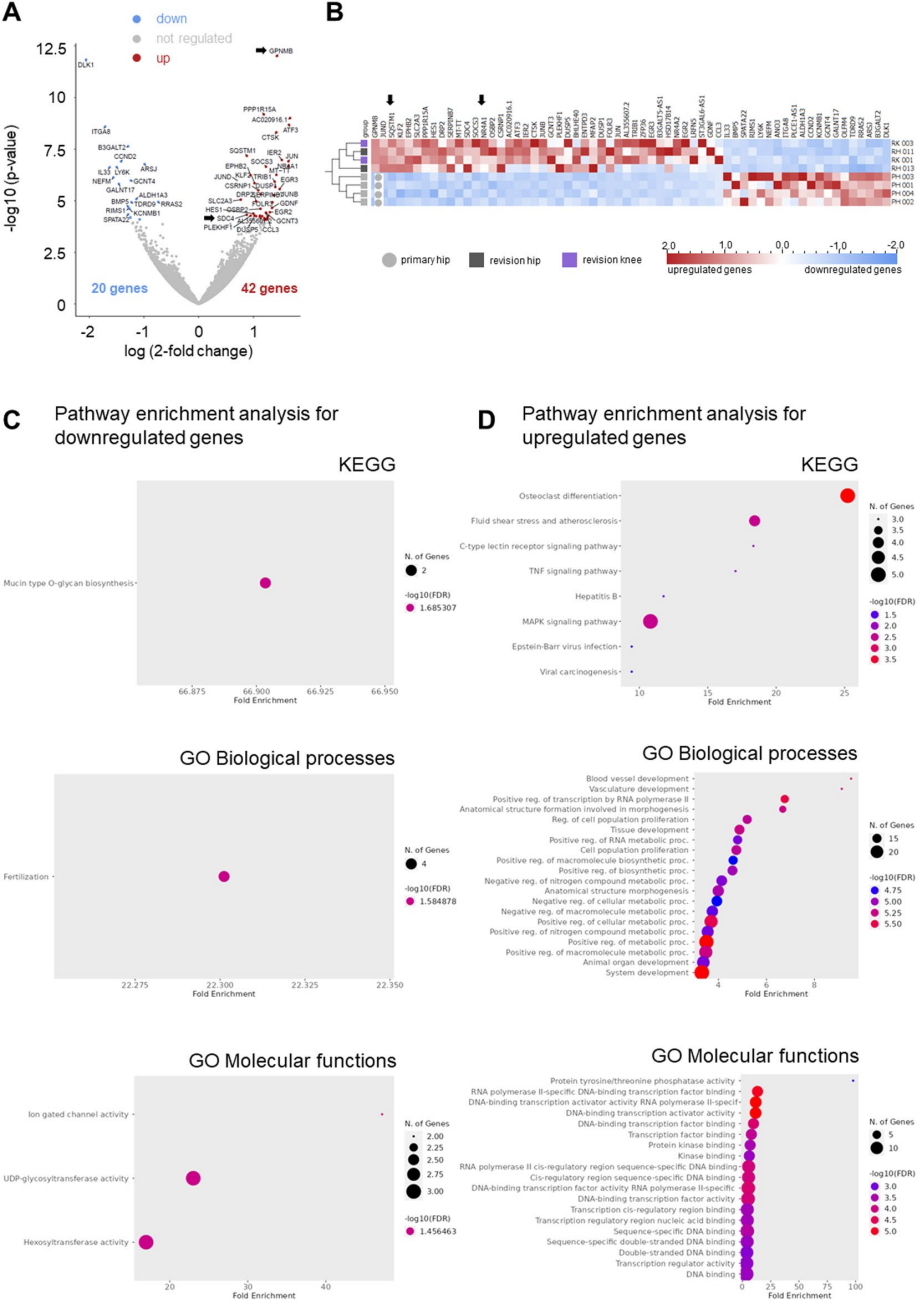
Transcriptome analysis of eight sex- and age-balanced BM-MSC preparations by RNA-seq. (**A**) Volcano plot emphasizing differentially expressed genes with DESeq2-based p_adj_-value cut-off 0.05. These genes were included in the gene set enrichment analysis in **C**,** D**. Arrows highlight *GPNMB* and *SDC4*. (**B**) Heatmap showing differentially expressed genes with DESeq2-based p_adj_-value cut-off 0.05. Qlucore Omics Explorer was used for the heatmap visualisation and normalization of the data (each column has mean of 0 and variance of 1) of four primary hip surgeries vs. four revisions (2x hip, 2x knee). Arrows highlight *GPNMB* and *SDC4*. (**C**) KEGG analyses (KEGG: https://www.kegg.jp/;^14^ of downregulated genes (upper panel) as identified using DESeq2. Gene ontologies for Biological Processes (middle panel) and Molecular Functions (lower panel) are included. (**D**) KEGG analyses of upregulated genes (upper panel) as identified using DESeq2. Gene ontologies for Biological Processes (middle panel) and Molecular Functions (lower panel) are included.

The first pilot sequencing run included RNA from the BM-MSCs of eight carefully age- and sex-matched patients as balanced discovery cohort: from 4 primary THA (PH, colour code: light grey: PH 001, PH 002, PH 003, PH 004), 2 revision THA (RH, colour code: dark grey: RH 011, RH 013), and 2 revision TKA (RK, colour code: violet: RK 001, RK 003). To uncover differentially expressed genes between the primary and revision samples, we utilized DEseq2 for differential gene expression analysis^12^visualised with a volcano plot (Fig. 2A, adjusted p-value ≤ 0.05). This analysis identified 62 significantly regulated genes, amongst which 42 genes exhibited higher and 20 lower expressions in revision samples (see data file submitted as supplemental information). A heatmap (Fig. 2B) illustrated the normalized expression of these genes. *GPNMB* was identified as the most prominent differentially expressed gene with > 3-fold upregulation and an adjusted *p*_adj_ value = 2.43E-08. Overall, high interindividual patient variability in transcriptome composition was observed.

Figure 2C shows analyses by the KEGG database including Gene Ontologies (GO terms) for Biological Processes and Molecular Functions for the 20 downregulated genes while Fig. 2D addresses the 42 upregulated genes. As to the downregulated genes, glycosylation of macromolecules, e.g. relevant for secretion, shows up as one major motif. The upregulated genes are relevant for a broad variety of processes, e.g. osteoclast differentiation and MAPK signalling, the development of different tissues as well as metabolic processes, and nucleic acid/transcription-associated processes.

### Relative GPNMB levels in bone marrow plasma and blood plasma are not different between primary and revision arthroplasties

In arthroplasty, identification of novel biomarkers to distinguish loosened implants from stably integrated implants is highly relevant. Thus, we decided to focus further investigations of potential biomarker candidates based on the following criteria: firstly, an upregulation in the revision cohorts and a notable gene expression as revealed by the base mean. The 12 upregulated genes with the highest base means (> 1000) were KLF2, BHLHE40, *NR4A1*, *PPP1R15A*, *IER2*, *CTSK*, *SOCS3*, *ZFP36*, *JUNB*, *DUSP1*, *GPNMB*, and *JUN*. Secondly, the occurrence of the encoded proteins in soluble forms (either secreted or by shedding of an extracellular domain) that can be quantitated by commercially available ELISA kits would be more advantageous than the choice of, e.g., intracellular transcription factors. This additional criterium was fulfilled by *GPNMB* within the 12 genes.

**Fig. 3:**
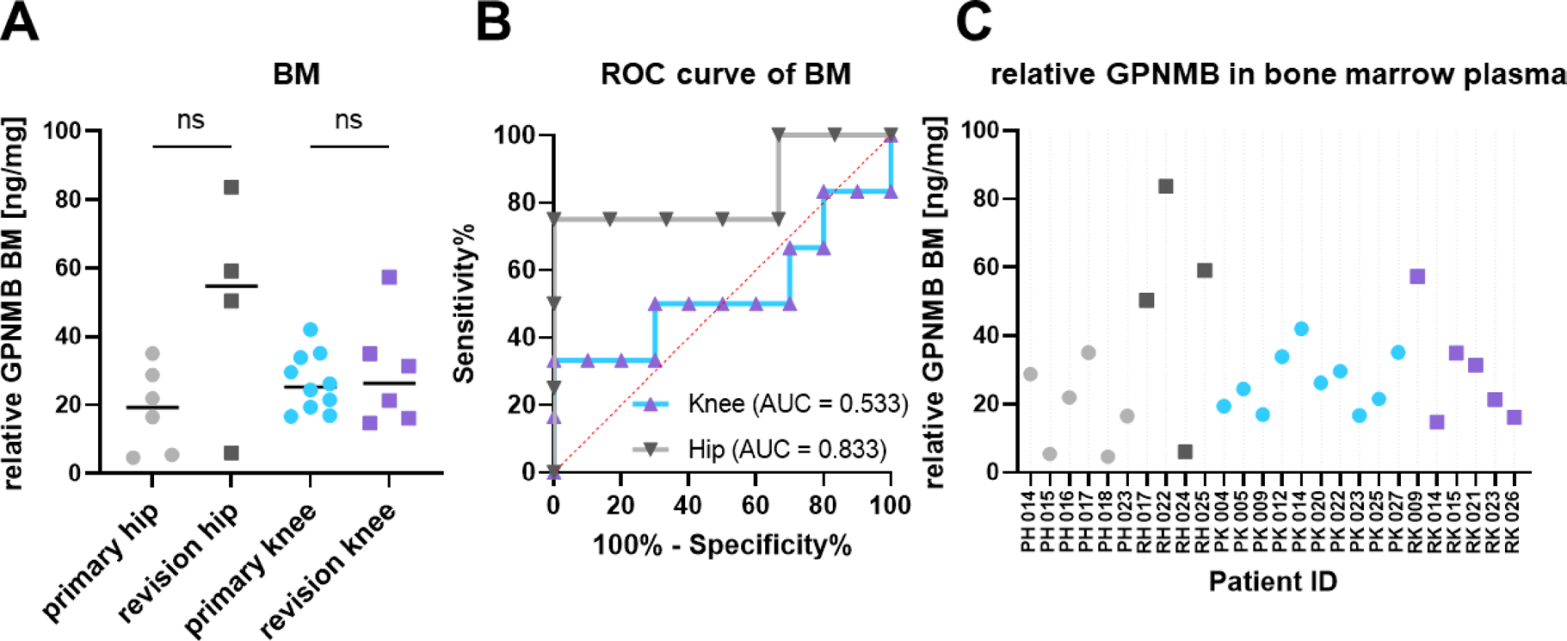
Relative levels of GPNMB in bone marrow plasma (BM). The absolute GPNMB levels detected in ELISA were normalised by the total protein concentration of the individual samples. (**A**) Relative GPNMB levels displayed via four patient cohorts. The black bar represents the median value. ns: not significant.(**B**) ROC curves (Receiver Operating Characteristics) provide an overview of the diagnostic quality based on bone marrow plasma levels of GPNMB measured by enzyme-linked immunosorbent assay for prediction of a revision sample. AUC: Area under the curve. (**C**) Relative GPNMB levels for each individual patient.

**Fig. 4:**
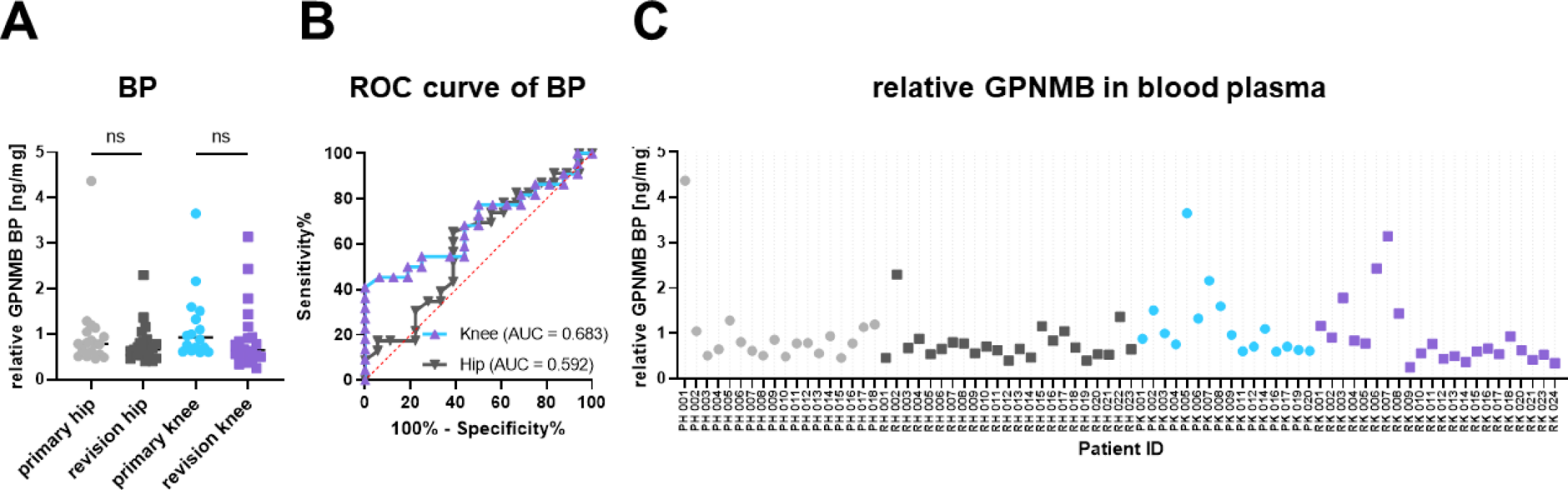
Relative levels of GPNMB in blood plasma (BP). The absolute GPNMB levels detected in ELISA were normalised by the total protein concentration of the individual samples. (**A**) Relative GPNMB levels displayed via four patient cohorts. The black bar represents the median value. ns: not significant. (**B**) Receiver operating characteristic curve (ROC) for prediction of a revision sample based on blood plasma levels of GPNMB measured by enzyme-linked immunosorbent assay. AUC: Area under the curve. (**C**) Relative GPNMB levels for each individual patient.

GPNMB, also called osteoactivin, is an ideal target matching premises for biomarkers. It is a type I transmembrane glycoprotein whose extracellular domain can be shedded from the cell surface after proteolytic cleavage and is secreted into body fluids^18^. Hence, we examined the soluble form of GPNMB in bone marrow and blood plasma of 26 or 79 patients, respectively. The concentration of soluble GPNMB determined by ELISA and total protein concentration allowed to calculate relative GPNMB concentrations. Figures 3 and 4 display the results for the four patient cohorts for bone marrow plasma and blood plasma, respectively. Overall, the relative concentrations are about tenfold higher in bone marrow plasma than in blood plasma. However, in both body fluids no statistically significant differences were detected between the four patient cohorts which is substantiated by the Receiver Operating Characteristic (ROC) curve analyses (panels B) in Figs. 3 and 4 which indicate that the GPNMB concentrations do not perform much better than a random selection. The red dashed line in the ROC curves (AUC of 0.5) indicates a random process.

### Relative GPNMB levels are significantly elevated in synovial fluid plasma at the time of knee revision arthroplasty

**Fig. 5:**
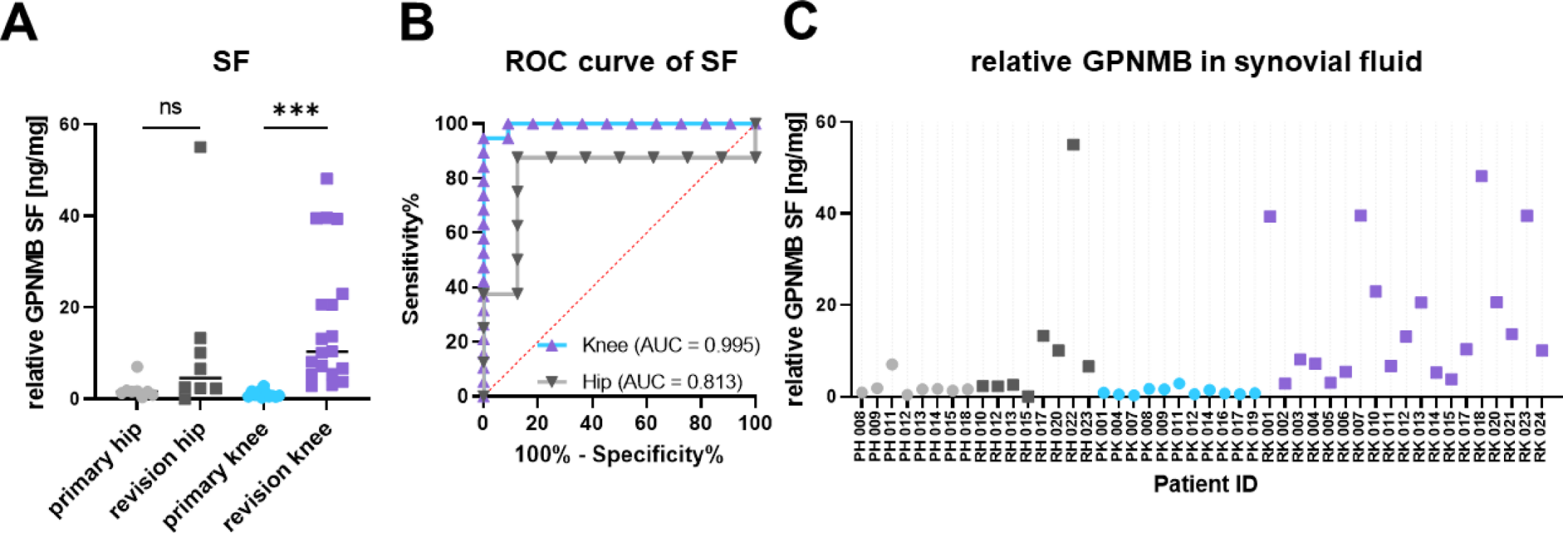
Relative levels of GPNMB in synovial fluid plasma (SF). The absolute GPNMB levels detected in ELISA were normalised by the total protein concentration of the individual samples. (**A**) Relative GPNMB levels displayed via four patient cohorts. The black bar represents the median value. ***p < 0.001, ****p < 0.0001, ns: not significant. (**B**) Receiver operating characteristic curve (ROC) for prediction of a revision sample based on synovial fluid plasma levels of GPNMB measured by enzyme-linked immunosorbent assay. AUC: Area under the curve. (**C**) Relative GPNMB levels for each individual patient.

The missing correlation of soluble GPNMB in bone marrow plasma with the RNA-seq data from BM-MSCs prompted additional considerations. Figure 1A visualises that both THA and TKA not only directly contact the bone marrow within the bony tissue but also the synovial fluid within the joint cavity, both resulting in extensive tissue-implant interfaces. We therefore decided to measure GPNMB levels in the synovial fluid samples as well. Figure 5A demonstrates statistically significant differences for revision knee vs. primary knee arthroplasty (*p* = 0.0002) while no significant differences were documented for revision hip vs. primary hip arthroplasty. The Receiver Operating Characteristic curve analysis in Fig. 5B indicates that the GPNMB concentration in synovial fluid of knee samples is able to predict the cohort membership with a high sensitivity and specificity. This shows the diagnostic quality of GPNMB as biomarker for knee arthroplasty (AUC = 0.995), performing better than a random selection. Contamination with blood, observed in a number of samples, is unlikely to interfere with the measured values due to the notably lower relative GPNMB concentrations in blood plasma than in synovial fluid plasma. This interpretation was confirmed by artificial admixture of blood into blood-free synovial fluid samples which resulted in comparable results, confirming absence of interference with ELISA performance by blood or haemolysis (data not shown).

The Spearman correlation was *r* = 0.4649 for the comparison between the relative GPNMB concentration in the synovial fluid and the summed roentgenographic defect length, indicating a moderate positive monotonic relationship between these variables, with statistical significance (*p* = 0.0449). No correlation was revealed with other roentgenographic parameters. For the knee revision surgeries, a moderate but significant correlation with the body mass index of the patients was found (*r* = 0.5607, *p* = 0.0125).

Due to the comparably low number of biosamples and the fact that most aseptic revision surgeries are classified histo-pathologically as mixtures of type I + type IV according to Krenn and Morawietz, not as distinct types, a potential correlation analysis between the relative GPNMB levels in synovial fluid and histo-pathological grading was not attempted.

Since GPNMB and SDC4 interact with each other as ligand and receptor^19^ the levels of SDC4 were determined in synovial fluid and bone marrow. The data is shown in Suppl. Fig. S2 and Suppl. Fig. S3. No statistically significant difference could be revealed between the four patient cohorts. Moreover, no correlation between relative GPNMB and relative SDC4 levels in both body fluids could be revealed.

### Total protein composition is similar in synovial fluid plasma of primary and revision surgeries

In order to ensure that the statistically different relative GPNMB levels in synovial fluid plasma are not due to massively altered protein composition, reducing SDS PAGE analysis was performed. The four cohorts PH, RH, PK and RK were checked for four stochastically chosen samples each. Suppl. Fig. S4 displays a total protein stain. Since synovial fluid is mainly an ultrafiltrate of blood and human serum albumin is one of the major blood proteins, with a nominal molecular weight of about 66,700 g/mol, the most prominent protein band observed in the study samples was interpreted as human serum albumin. Moreover, two prominent bands at about 50,000 and 25,000 g/mol hint at the presence of immunoglobulins (heavy and light chains, respectively), which are also abundant in blood plasma. The total protein patterns are similar within and between the patient cohorts. This confirms the notion that after arthroplasty, a neo-synovial (also called periprosthetic) membrane is formed, with comparable properties to a native synovial membrane. In particular, the overall protein composition is retained.

### The GPNMB extracellular domain is present in all three body fluid plasmas

**Fig. 6:**
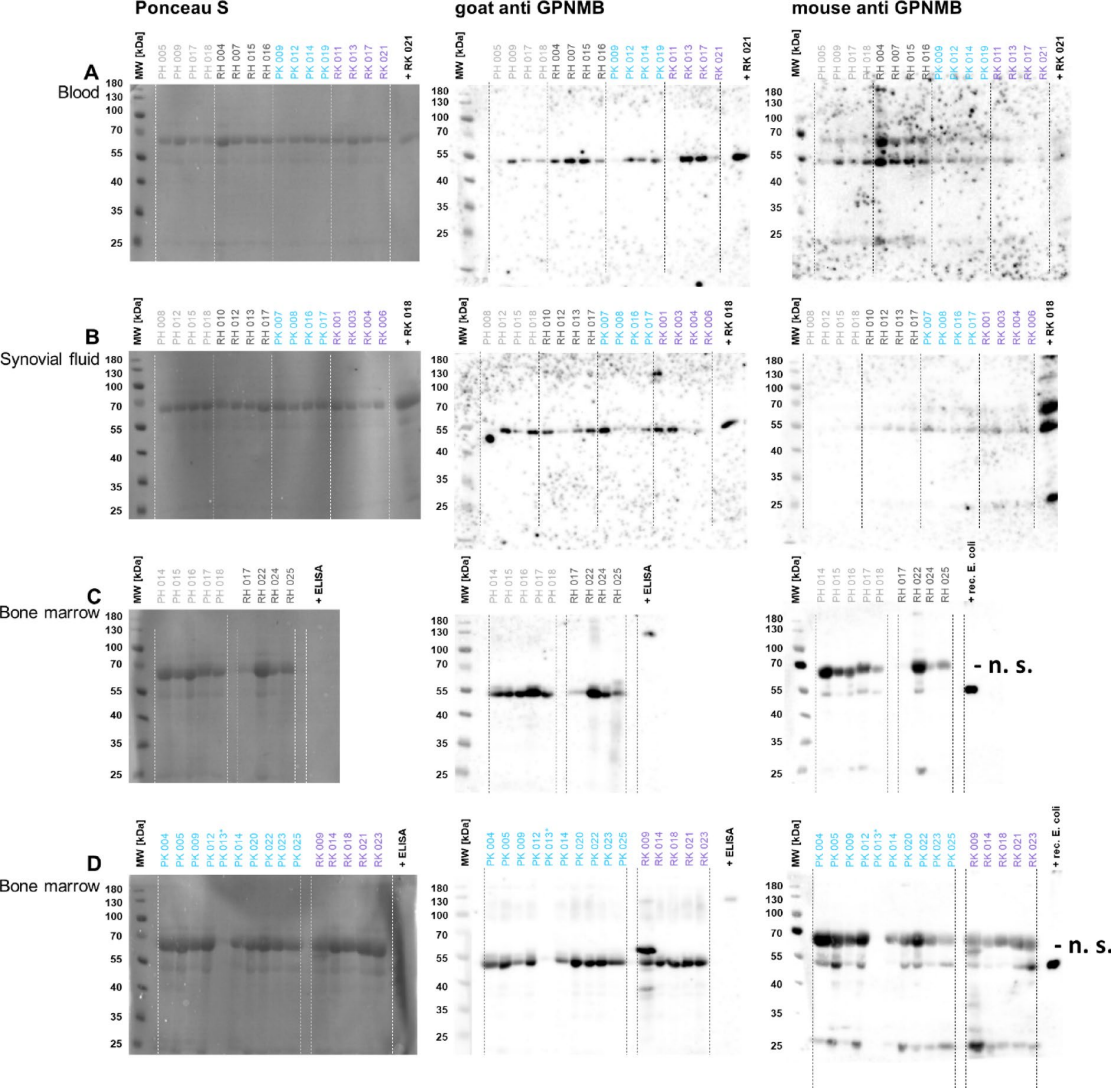
Total protein stain (left panels) and Western blot for GPNMB (middle and right panels, different primary antibodies). - n. s.: non-specific reactions of mouse antibody. Guidance lines are included for the readers´ convenience. All lanes loaded with samples are displayed. Completely uncropped original blots are included in the Supp. Material. (**A**) Randomly chosen blood plasma samples from the four patient cohorts. (**B**) Randomly chosen synovial fluid plasma samples from the four patient cohorts. (**C**) All bone marrow plasma samples from the four patient cohorts.

To confirm the identity of GPNMB in the plasma samples, two different antibodies were used. As positive controls, the recombinant ELISA standard protein, a commercially available *E. coli*-expressed recombinant GPNMB (cf. 2.13.) or bone marrow plasma for endogenous GPNMB were used. Full-length variants of endogenous human GPNMB have been observed with 115,000 and 90,000 g/mol^16^. The extracellular domain of GPNMB (amino acids 1–486) has been reported with an apparent molecular weight of 52,000 g/mol, which depends on posttranslational modifications. Figure 6 displays that the positive controls are successfully detected by both antibodies. In the patient samples, both antibodies detected GPNMB species at 52,000 g/mol i.e. most likely the extracellular domain while full-length species are largely absent. In blood plasma and synovial fluid plasma, the protein bands were focused and distinct (Fig. 6A, B). In bone marrow plasma (Fig. 6C, D), the exact migration position of the protein species slightly varied between samples. Some protein bands revealed notable molecular weight heterogeneity. Bone marrow samples clearly demonstrated the highest GPNMB levels in relation to total protein, confirming the ELISA data. The murine GPNMB antibody performed less well than the goat antibody, due to cross-reaction with protein species at about 67,000 g/mol (probably albumin), most prominently seen with bone marrow plasma (Fig. 6C, D: indicated by “- n. s.”).

### RNA-seq analysis of an extended patient cohort including arthrofibrosis confirms the role of GPNMB

The significantly elevated relative GPNMB protein levels in synovial fluid plasma prompted us to seek for extended information in the BM-MSCs. To do so, we subjected RNA from BM-MSCs of additionally 20 patients to RNA-seq, including five patients with arthrofibrosis at the knee as diagnosed by clinical symptoms, i.e. with type IV implant failure as the primary cause for revision surgery.

**Fig. 7:**
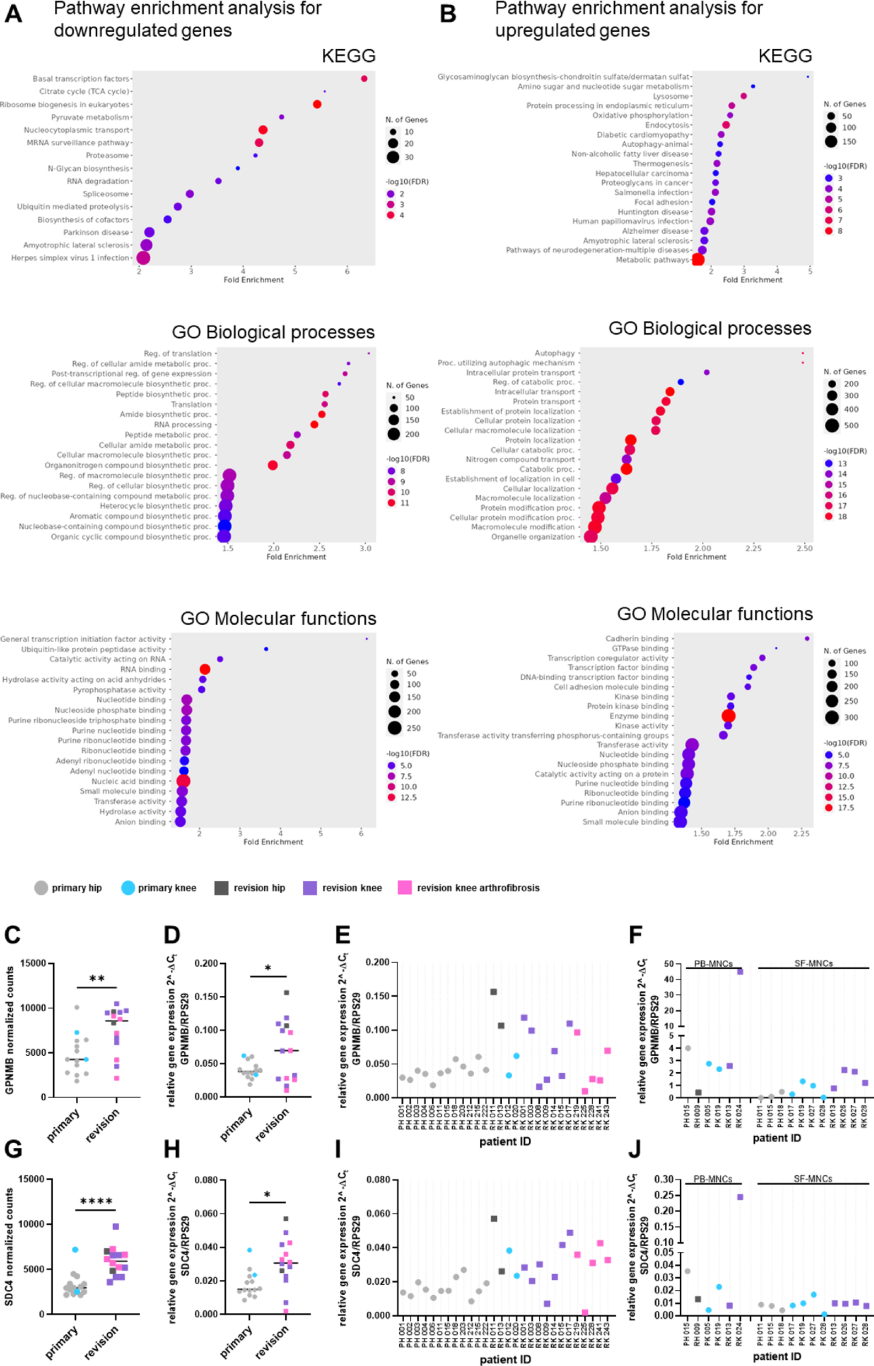
Transcriptome analysis of all primary vs. all revision arthroplasties at both implantation sites (28 samples). (**A**) KEGG analyses (KEGG: https://www.kegg.jp/;^14^ of downregulated genes (upper panel). Gene ontologies for Biological Processes (middle panel) and Molecular Functions (lower panel) are included. (**B**) KEGG analyses of upregulated genes (upper panel). Gene ontologies for Biological Processes (middle panel) and Molecular Functions (lower panel) are included. (**C**) RNA-seq data for *GPNMB* in BM-MSCs (normalised counts). For the statistical comparisons between primary and revision the parametric unpaired Welch’s t-test was used. Experimental data are shown as individual values. (**D**,** E**) *GPNMB* relative expression levels in BM-MSCs based on *RPS29* as housekeeping gene. **E** indicates the levels for individual samples. (**F**) *GPNMB* relative expression levels in MNCs from blood (PB) and synovial fluid (SF). (**G**) RNA-seq data for *SDC4* in BM-MSCs (normalised counts). For the statistical comparisons between primary and revision the parametric unpaired Welch’s t-test was used. Experimental data are shown as individual values. **(H**,** I)** *SDC4* relative expression levels in BM-MSCs based on *RPS29* as housekeeping gene. **I** indicates the levels for individual samples. (**J**) *SDC4* relative expression levels in MNCs from blood (PB) and synovial fluid (SF). The black bar in C, D, G, H represents the median value. *p < 0.05, **p < 0.01, ***p < 0.001, ****p < 0.0001.

Consequently, the complete cohort of 28 patients investigated within two technically independent sequencing runs comprised 12 primary THA (PH, colour code: light grey), 2 revision THA (RH, colour code: dark grey), 2 primary TKA (PK, colour code: blue) and 12 revision TKA (7 with type I and type IV aseptic implant loosening, colour code: violet plus 5 with arthrofibrosis, colour code: pink). The high patient variability in transcriptome composition reported from the first sequencing run was confirmed. Differentially expressed genes between all 14 primary surgery samples were compared with all 14 revision surgery samples using DESeq2 (adjusted p-value ≤ 0.05) while controlling for sequencing runs, patient age and sex. This served to identify major pathways, which are different between primary and revision surgery but operative in both anatomical sites. 2908 DEGs were identified (documented in the data file submitted as supplemental information). 2033 genes were upregulated, 875 genes were downregulated. Since BM-MSCs play a significant role in arthroplasty, particularly in the context of joint repair and regeneration, understanding the biological consequences based on these large number of DEGs may provide insights into underlying mechanisms of arthroplasty failure. Figure 7A displays analyses by the KEGG database including GO terms for Biological Processes and Molecular Functions for the downregulated genes while Fig. 7B addresses the upregulated genes. We observed a more robust functional pathway enrichment in the complete cohort compared to the small cohort (Fig. 2C, D: the robustness is indicated by the False Discovery Rate (FDR)), due to the higher number of DEGs identified in the complete cohort (2908 DEGs) versus the discovery cohort (62 DEGs). While downregulated genes in revision samples are involved in transcription (Fig. 7A: KEGG and GO Molecular Function) and translation (Fig. 7A: GO Biological Process), upregulated genes participate in regulation of autophagy (Fig. 7B: GO Biological Process) and glycosaminoglycan biosynthesis (Fig. 7B: KEGG). These data are interesting, because autophagy is a fundamental cell survival mechanism that allows cells to adapt to metabolic stress through the degradation and recycling of intracellular components to generate macromolecular precursors and produce energy^20^. Furthermore, malfunction of glycosaminoglycan biosynthesis underlies a range of severe disorders, frequently affecting skeletal development and cognitive functions^21^.

Within the 2908 DEGs, *GPNMB* was significantly upregulated in the revision samples as documented in the normalized counts from RNA-seq and the relative gene expression levels from qRT-PCR (Fig. 7C–E). Compared to the pilot cohort, *GPNMB* is less prominent. *SDC4*, the ligand for GPNMB protein, was highly significantly upregulated (Fig. 7G–I) while the data on protein level do not support a role as a soluble biomarker.

According to reports from the literature, BM-MSCs might not be the prime source of GPNMB but monocytes/macrophages, instead^22^. Therefore, qRT-PCR was performed on MNCs that were retrieved from synovial fluid and blood, respectively (no cells were available from bone marrow since the entire MNCs were used for culture development into BM-MSCs). The relative gene expression levels were notably higher in MNCs than in BM-MSCs (Fig. 7F vs. Fig. 7E). The relative gene expression levels of *SDC4* were comparable in MNCs and BM-MSCs (Fig. 7J vs. Fig.7I).

### GPNMB RNA expression levels in BM-MSCs and GPNMB protein levels in synovial fluid plasma are not correlated

The complete set of body materials was available only for 5 patients (see Suppl. Table 1). Only 10 patients were available with paired data sets from RNA-seq of BM-MSCs and synovial fluid plasma: 3x PH, 1x RH, 1x PK, and 5x RK. The results of the correlation analysis are displayed in Suppl. Fig. S5. The Spearman coefficient was *r* = 0.4788 pointing towards a moderate degree of correlation, with *p* = 0.1663 i.e. not significant.

## Discussion

### The need for biomarkers for implant loosening and failure

In arthroplasty, it is of prime importance to reliably identify loosened implants from stably integrated implants, which are symptomatic for other reasons, in order to select the optimal treatment regimen. This is currently not a straightforward task, based on retrospective roentgenographic analyses combined with analyses of bacterial infiltration or a histological analysis of intraoperatively removed tissue. The extended roentgenographic technique that was performed in the present study specifically emphasises the precise measurement of the length of radiolucent lines along the corresponding interface^9,10^. The finding that the defect length was statistically significant while the number of defect zones was not can be explained by the variable size of the prostheses: “small” sled, “medium-sized” full dentures or “large” revision prostheses. All these prostheses were either loose in different areas or there were clear signs of loosening combined with inconclusive areas which were not measured.

Genes or proteins are favourable alternatives to roentgenography, microbiology and histology since they can be quantified in labs, which do not have access to specialised equipment and personnel. In the past, several studies identified potential biomarkers with elevated levels in synovial fluid or blood serum of patients with aseptic implant loosening: “Soluble B- and T-lymphocyte attenuator”^23^ CHIT1^24^, CCL18^24^, IL-6 and CRP^25^ osteoprotegerin, receptor activator of nuclear factor-kappa B ligand, cross-linked N-terminal telopeptide, tumour necrosis factor-alpha, and interleukin-1β^26^.

### High benefit of GPNMB as biomarker for implant loosening and failure

GPNMB has up to the present day not been addressed as a biomarker in the musculoskeletal system and for endoprostheses. However, the value of GPNMB as a biomarker has already been the subject of several studies. GPNMB protein levels were found to be elevated in human cerebrospinal fluid in neurological diseases^27–29^. GPNMB blood plasma levels were elevated in lysosomal storage diseases^30^, in visceral acid sphingomyelinase deficiency^31^, in diabetes mellitus-related cataract^32^, Cystic Fibrosis^33^, alcohol-associated hepatitis^34^, and in non-alcoholic steatohepatitis^35^. Lower levels of GPNMB in blood plasma were observed in patients with heart failure compared to controls without heart failure^36^. GPNMB was described as a urinary biomarker for chronic kidney disease, with a significantly elevated GPNMB/creatinine ratio compared to healthy controls^37^.

In the study presented here, GPNMB was identified based on its RNA levels in BM-MSCs of eight patients, upregulated in four revision cases. Due to high interindividual variability in gene expressions, in the extended data set of 28 patients there was one sample from a primary surgery with GPNMB expression higher than the median in revision surgeries while three patients from revision surgeries showed GPNMB levels comparable or lower than the median in primary surgeries. The data from RNA-seq of the large cohort is interesting, pinpointing autophagy and malfunction of glycosaminoglycan biosynthesis^20,21^. These data suggest a cellular adaptation of BM-MSCs in revision samples and indicate that the dysregulation of autophagy and glycosaminoglycans may contribute to the development of arthroplasty failure. The biological relevance of these processes for, e.g., the capacity of BM-MSCs to differentiate into osteoblasts and chondrocytes remains to be determined.

The current method for derivation of MSCs relies on their selection from MNCs through plastic adhesion, taking into account culture-induced changes that make them different from their in vivo-counterparts. It is surprising that in the study presented here, GPNMB was initially identified from RNA-seq of BM-MSCs which was then confirmed and substantiated on the protein level in synovial fluid i.e. in a body fluid that is in tight contact with the implant, retrieved from the patients without notable alterations of the in vivo-situation. Inasmuch MNCs from bone marrow and synovial fluid from the same human donors share molecular properties and whether, actually, MNCs from synovial fluid would be the major producers of GPNMB, needs to be investigated in future studies. Our pilot data on relative gene expression levels were notably higher for MNCs from peripheral blood and synovial fluid than for BM-MSCs, suggesting a MNCs/monocyte-derived macrophages origin^22^.

Our data does not give any clues to a statistically significant elevation of GPNMB in synovial fluid plasma of revision hip arthroplasty patients, contrasting with revision knee arthroplasty. This may question its diagnostic potential. The differences observed could possibly be explained by a mechanism related to the contrasting biomechanical conditions at the knee and hip joints. In knee arthroplasties, multidirectional relative motions are typically observed, i.e., axial displacement, rotation, and in particular tipping of the tibial component under load cycles^38,39^. These movements cause cyclical volume changes in the interface gap^40^. In hip prostheses, axial and torsional micromotions are the most common type, typically along the stem–bone interface or acetabular margin^41^. These motion patterns do not result in significant volume changes in the interface, thus limiting active fluid exchange^42^.

The techniques for determination of GPNMB levels (ELISA, total protein) are easy to perform and comparably fast. An additional benefit of the use of GPNMB in implant failure results from the fact that synovial fluid is a body fluid that is routinely taken already pre-operatively by diagnostic joint puncture from patients who complain about malfunctions of their implants. On the contrary, a biomarker in bone marrow would require more invasive procedures for the marrow harvest. Future efforts might be dedicated to the comparison of the performance of GPNMB with factors highlighted as biomarkers in previous studies, to additional diagnoses, or to the development of more rapid methods of quantification including implant-borne sensors.

## Conclusion

GPNMB has the potential to act as a prospective and quantitative parameter to assess different stages of implant loosening. Radiography and histo-pathological classification of the periprosthetic membrane, on the contrary, are used retrospectively for diagnosis and are subjective measures, performed mainly at the end of a full loosening process.

The present study did not assess the relative levels of GPNMB in cases of septic implant loss, though. This will be the subject of future studies, which will include the prospective sampling of relevant biomaterial since in cases of implant failure, it is imperative to reliably differentiate between low-grade periprosthetic joint infections, septic and aseptic causes. The control group enrolled patients without arthroplasty, i.e. native joints without implant. This pertains to the difficulties in recruiting patients with radiographically fixed but nevertheless loosened implants like patients with periprosthetic fractures or pathologies other than loosening. This challenge amplifies with the need for higher patient numbers as they are required for statistical analyses. Also, patients with periprosthetic joint infections were not included. Overall, the notable differences between the patient samples pinpoint the need for novel strategies in personalised medicine.

## Supplementary Information

Below is the link to the electronic supplementary material.

Supplementary Material 1

Supplementary Material 2

## Data Availability

The bulk RNA-seq data generated and analysed during the current study are available in the Gene Expression Omnibus (GEO) (NCBI) repository under accession number GSE276597.

## Abbreviations

AUC: Area under the curve
BM: MSC Bone-marrow derived mesenchymal stromal cell
ELISA: Enzyme-linked immunosorbent assay
FDR: False discovery rate
GPNMB: Glycoprotein non-metastatic protein B
KSRESS: Knee society radiographic evaluation system
MNC: Mononuclear cell
PH: Primary hip arthroplasty
PK: Primary knee arthroplasty
RH: Revision hip arthroplasty
RK: Revision knee arthroplasty
RNA-seq: Ribnonucleic acid sequencing
ROC: Receiver Operating Characteristic
THA: Total hip arthroplasty
TKA: Total knee arthroplasty
