# Supplementary material for: Glycoprotein non-metastatic melanoma protein B is a potential biomarker for arthroplasty aseptic loosening
Source: Sci Rep. 2025 Sep 12;15:32419. doi: 10.1038/s41598-025-13922-3 (PMC12432123; doi:10.1038/s41598-025-13922-3)
Supplement: Supplementary file 1 — Supplementary Material 1 [file 41598_2025_13922_MOESM1_ESM.docx]

Supplementary Information

**Glycoprotein non-metastatic melanoma protein B is a potential biomarker for arthroplasty aseptic loosening stages**

Patrik Schadzek^1,7†^, Alexander Derksen^2†^, Wiebke Behrens^3,7†^, Maike Kosanke^4^, Oliver Dittrich-Breiholz^4^, Anika Hamm^1,7^, Kirsten Elger^1,7^, Yvonne Roger^1,7^, Ines Yang^3,7^, Meike Stiesch^3,7^, Yvonne Noll^2^, Marco Haertlé^2^, Lars-René Tuecking^2^, Christina Stukenborg-Colsman^2^, Henning Windhagen^2,7^, Doan Duy Hai Tran^5,8^, Anette Melk^6^, Andrea Hoffmann ^1,7*^

^1^ Hannover Medical School, Department of Orthopaedic Surgery, Biological Basics for Biohybrid Implants, Anna-von-Borries-Str. 1-7, 30625 Hannover, Germany

^2^ Hannover Medical School and DIAKOVERE Annastift, Department of Orthopaedic Surgery, Anna-von-Borries-Str. 1-7, 30625 Hannover, Germany

^3^ Hannover Medical School, Department of Prosthetic Dentistry and Biomedical Materials Science, Carl-Neuberg-Str. 1, 30625 Hannover, Germany

^4^ Hannover Medical School, Research Core Unit Genomics, Carl-Neuberg-Str. 1, 30625 Hannover, Germany

^5^ University Medical Centre Göttingen, Department of Nephrology and Rheumatology, Robert-Koch-Str. 40, 37075 Göttingen, Germany

^6^ Hannover Medical School, Department of Paediatric Kidney, Liver and Metabolic Diseases, Carl-Neuberg-Str. 1, 30625 Hannover, Germany

^7^ Lower Saxony Centre for Biomedical Engineering, Implant Research and Development (NIFE), Stadtfelddamm 34, 30625 Hannover, Germany

^8^ DZHK German Centre for Cardiovascular Research, Partner Site Lower Saxony, Germany

^†^ contributed equally

^*^ corresponding author: [hoffmann.andrea@mh-hannover.de](mailto:hoffmann.andrea@mh-hannover.de), tel. ++49 511 532 1442, Hannover Medical School, Department of Orthopaedic Surgery, Biological Basics for Biohybrid Implants OE 8893, Stadtfelddamm 34, D-30625 Hannover, Germany

**Supplementary Figure S1**


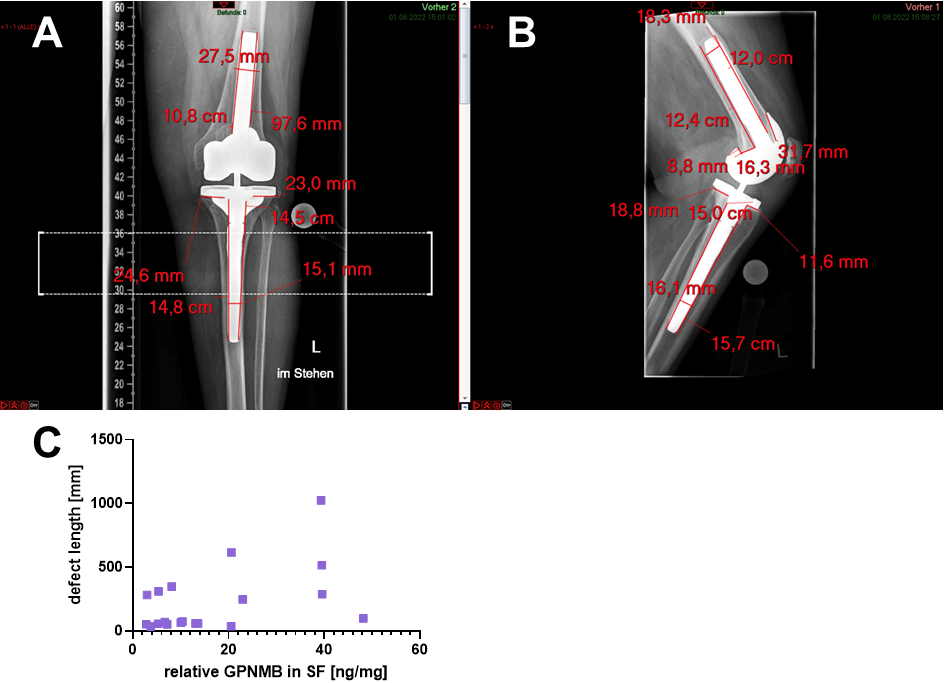


**Suppl. Fig. S1.** Correlation analysis of roentgenographic defect length and relative GPNMB levels in synovial fluid.

(**A, B**) X-rays in two planes of a total knee arthroplasty with measurement of the radiolucent lines and calculation of defect length.

(**C**) Spearman correlation analysis of defect length (in mm) versus relative GPNMB levels in synovial fluid.

**Supplementary Figure S2**

**
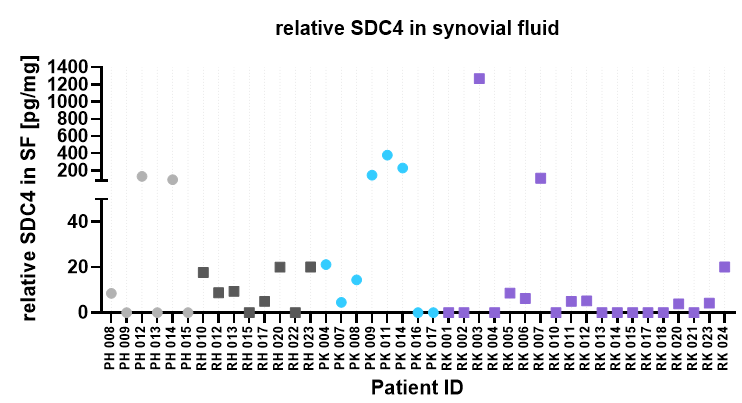
**

**Suppl. Fig. S2.** Total protein stain for four randomly chosen synovial fluid plasma samples from all four patient cohorts.

**Supplementary Figure S3**

**
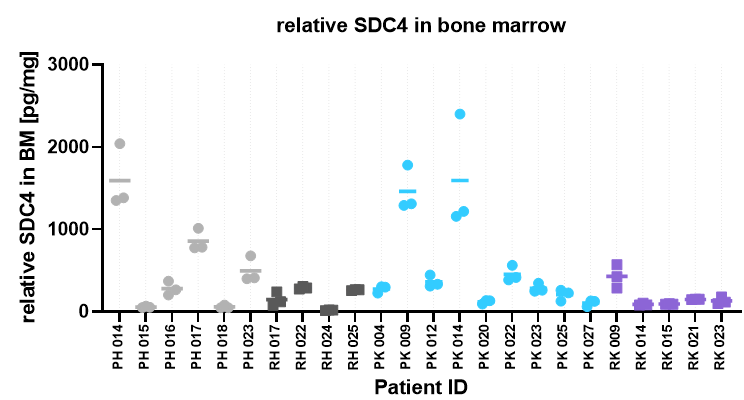
**

**Suppl. Fig. S3.** Relative levels of SDC4 in synovial fluid plasma (SF). The absolute SCD4 levels detected in ELISA were normalised by the total protein concentration of the individual samples. Relative SCD4 levels for each individual patient are shown.

**Supplementary Figure S4**

**
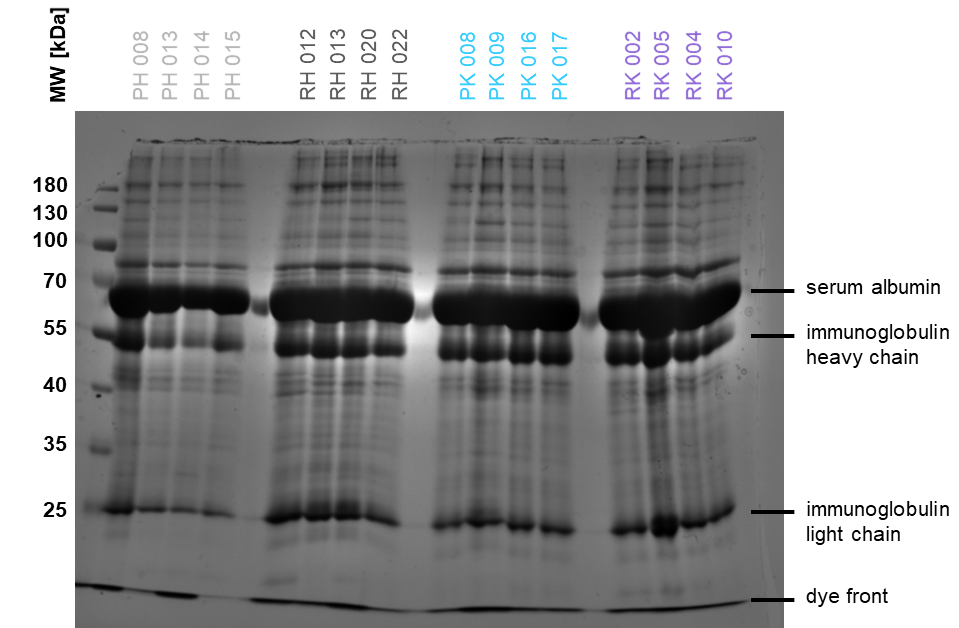
**

**Suppl. Fig. S4.** Relative levels of SDC4 in bone marrow plasma (BM, three individual aliquots per patient). The absolute SCD4 levels detected in ELISA were normalised by the total protein concentration of the individual samples. Relative SCD4 levels for each individual patient are shown. All lanes loaded with samples are displayed. The completely uncropped original gel is included as Suppl. Fig. S8.

**Supplementary Figure S5**

**
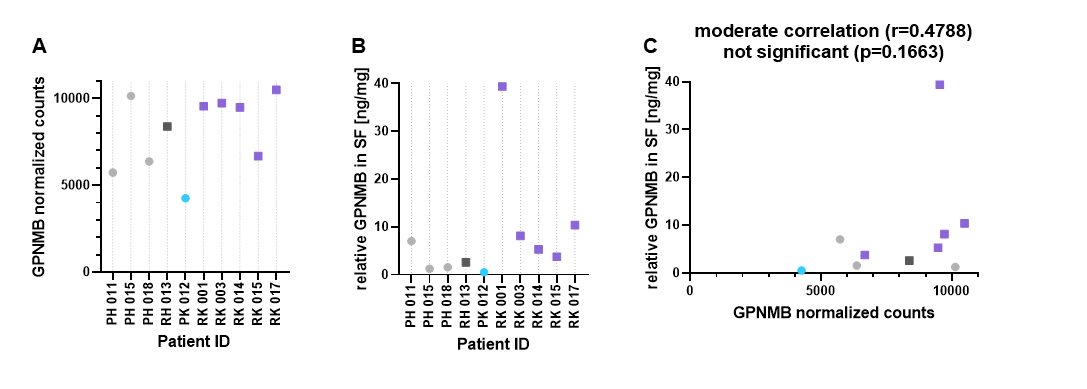
**

**Suppl. Fig. S5.** Correlation analysis between RNA expression levels in BM-MSCs and relative protein levels in synovial fluid plasma for GPNMB in the 10 available patients sample pairs.

(**A**) RNA-seq data normalised counts for *GPNMB*.

(**B**) Relative protein levels for GPNMB in synovial fluid plasma.

(**C**) Spearman correlation analysis.

**Supplementary Figure S6 displaying entire gel (related to Fig. 6 main text body)**

**
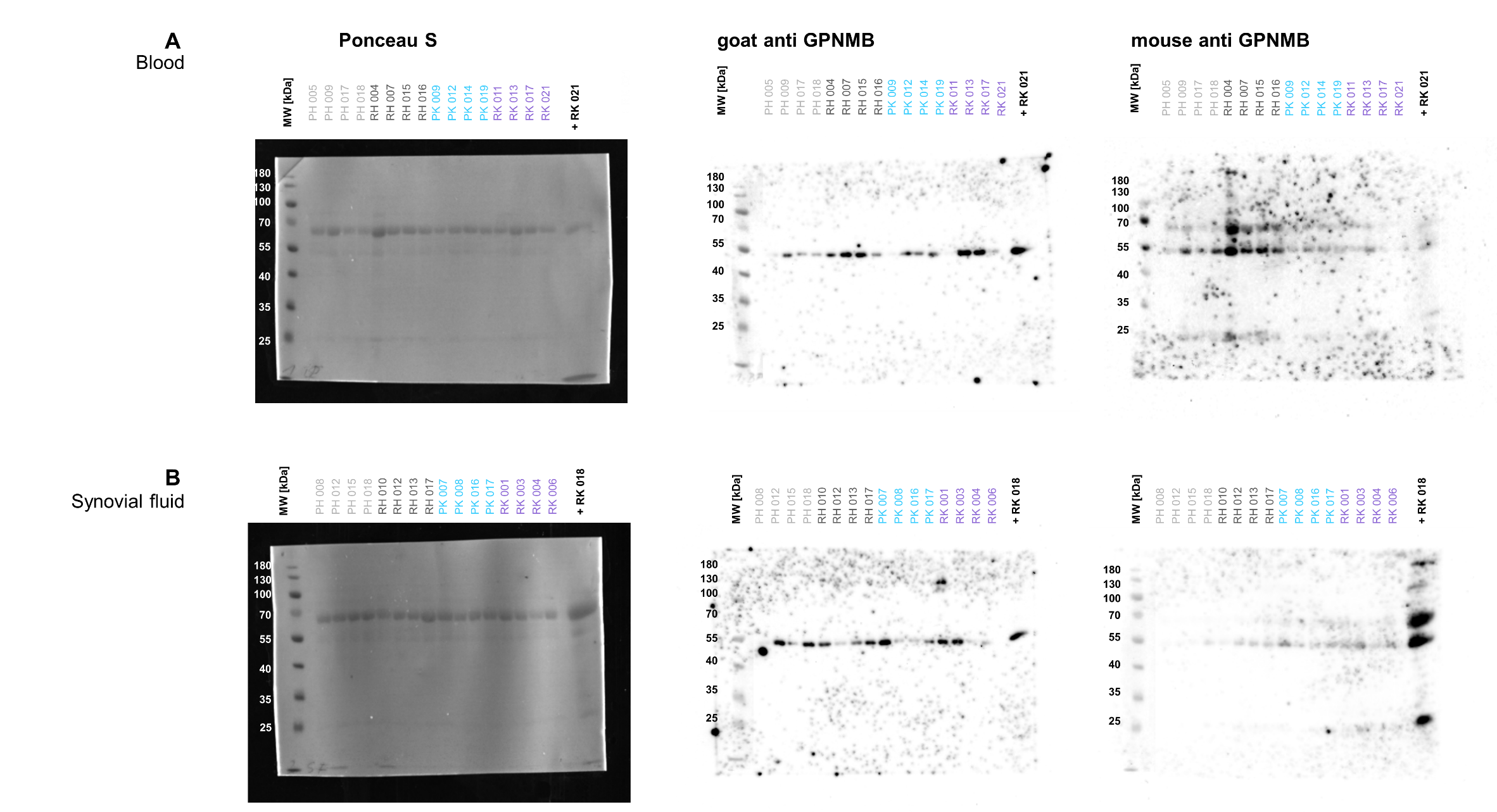
**

**Supplementary Figure S7 displaying entire gel (related to Fig. 6 main text body)**

**
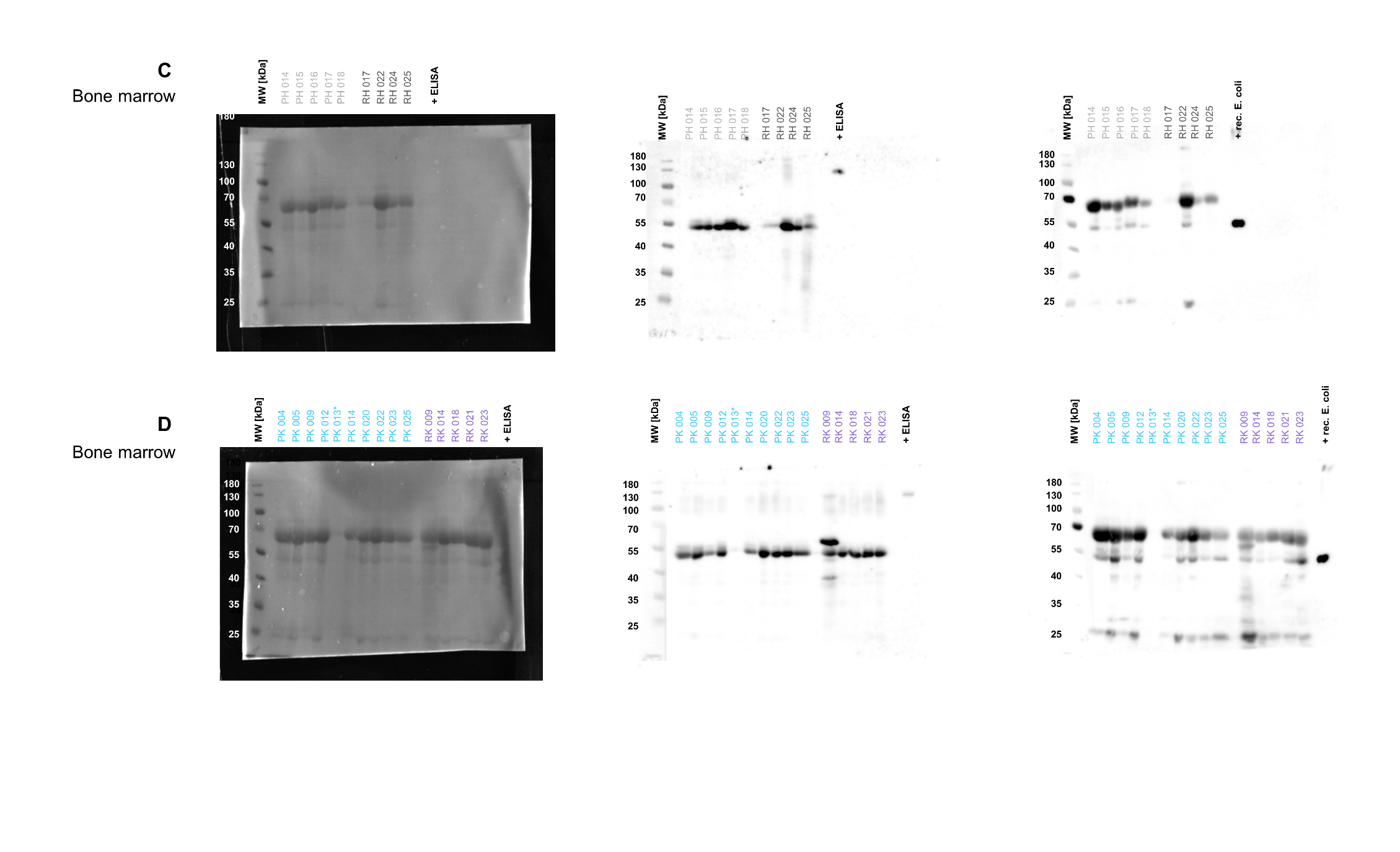
**

**Supplementary Figure S8 displaying entire gel (related to Fig. S4)**


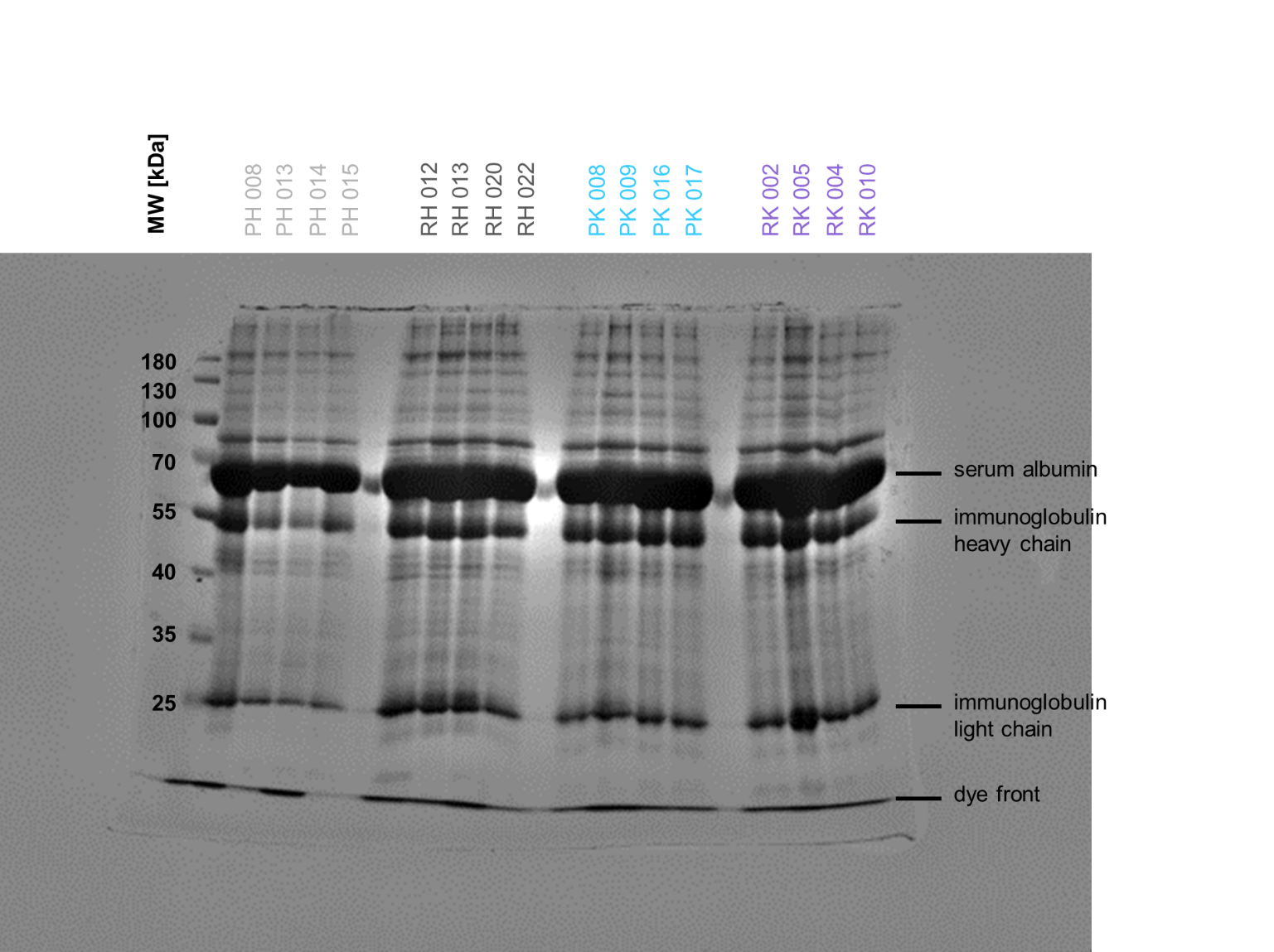


**Suppl. Table 1: Patient information for MSC RNA sequencing and for ELISA samples.**

| **Patient ID** | **Patient age (years)** | **Patient gender**  **(m = male,**  **f = female)** | **Body mass index** | **BM-MSCs RNA sequencing**  **(+ = yes,**  **- = no)** | **Bone marrow plasma**  **(+ = yes,**  **- = no)** | **Blood**  **(+ = yes,**  **- = no)** | **Synovial fluid**  **(+ = yes,**  **- = no)** |
| --- | --- | --- | --- | --- | --- | --- | --- |
| **PH 001** | **66** | **f** | **22.1** | **+** | **-** | **+** | **-** |
| **PH 002** | **61** | **f** | **25.2** | **+** | **-** | **+** | **-** |
| **PH 003** | **82** | **f** | **26.9** | **+** | **-** | **+** | **-** |
| **PH 004** | **67** | **m** | **29.7** | **+** | **-** | **+** | **-** |
| **PH 005** | **62** | **f** | **27.7** | **-** | **-** | **+** | **-** |
| **PH 006** | **64** | **m** | **32.1** | **+** | **-** | **+** | **-** |
| **PH 007** | **57** | **f** | **25.2** | **-** | **-** | **+** | **-** |
| **PH 008** | **86** | **f** | **22.7** | **-** | **-** | **+** | **+** |
| **PH 009** | **83** | **m** | **24.8** | **-** | **-** | **+** | **+** |
| **PH 010** | **74** | **f** | **28.1** | **-** | **-** | **+** | **-** |
| **PH 011** | **70** | **f** | **33.2** | **+** | **-** | **+** | **+** |
| **PH 012** | **55** | **m** | **27.8** | **-** | **-** | **+** | **+** |
| **PH 013** | **71** | **f** | **22.2** | **-** | **-** | **+** | **+** |
| **PH 014** | **82** | **f** | **24.8** | **-** | **+** | **+** | **+** |
| **PH 015** | **82** | **m** | **30.4** | **+** | **+** | **+** | **+** |
| **PH 016** | **59** | **f** | **34.2** | **-** | **+** | **+** | **-** |
| **PH 017** | **62** | **f** | **32.3** | **-** | **+** | **+** | **-** |
| **PH 018** | **82** | **f** | **25.7** | **+** | **+** | **+** | **+** |
| **PH 023** | **70** | **m** | **31.4** | **-** | **+** | **-** | **-** |
| **PH 203** | **61** | **f** | **34.9** | **+** | **-** | **-** | **-** |
| **PH 212** | **67** | **f** | **23.0** | **+** | **-** | **-** | **-** |
| **PH 215** | **79** | **m** | **31.0** | **+** | **-** | **-** | **-** |
| **PH 222** | **62** | **f** | **27.6** | **+** | **-** | **-** | **-** |
| **RH 001** | **81** | **m** | **25.5** | **-** | **-** | **+** | **-** |
| **RH 002** | **22** | **f** | **21.1** | **-** | **-** | **+** | **-** |
| **RH 003** | **82** | **f** | **25.2** | **-** | **-** | **+** | **-** |
| **RH 004** | **83** | **f** | **23.9** | **-** | **-** | **+** | **-** |
| **RH 005** | **64** | **m** | **26.6** | **-** | **-** | **+** | **-** |
| **RH 006** | **60** | **m** | **27.8** | **-** | **-** | **+** | **-** |
| **RH 007** | **32** | **m** | **20.0** | **-** | **-** | **+** | **-** |
| **RH 008** | **75** | **f** | **20.7** | **-** | **-** | **+** | **-** |
| **RH 009** | **74** | **f** | **25.2** | **-** | **-** | **+** | **-** |
| **RH 010** | **73** | **f** | **20.8** | **-** | **-** | **+** | **+** |
| **RH 011** | **69** | **f** | **21.8** | **+** | **-** | **+** | **-** |
| **RH 012** | **64** | **m** | **32.6** | **-** | **-** | **+** | **+** |
| **RH 013** | **68** | **f** | **27.8** | **+** | **-** | **+** | **+** |
| **RH 014** | **67** | **f** | **28.7** | **-** | **-** | **+** | **-** |
| **RH 015** | **78** | **m** | **25.7** | **-** | **-** | **+** | **+** |
| **RH 016** | **62** | **m** | **21.9** | **-** | **-** | **+** | **-** |
| **RH 017** | **73** | **f** | **29.2** | **-** | **+** | **+** | **+** |
| **RH 018** | **72** | **m** | **51.9** | **-** | **-** | **+** | **-** |
| **RH 019** | **62** | **m** | **37.6** | **-** | **-** | **+** | **-** |
| **RH 020** | **76** | **m** | **25.8** | **-** | **-** | **+** | **+** |
| **RH 021** | **63** | **m** | **35.6** | **-** | **-** | **+** | **-** |
| **RH 022** | **82** | **m** | **28.9** | **-** | **+** | **+** | **+** |
| **RH 023** | **41** | **f** | **22.3** | **-** | **-** | **+** | **+** |
| **RH 024** | **80** | **f** | **26.4** | **-** | **+** | **+** | **-** |
| **RH 025** | **76** | **m** | **26.2** | **-** | **+** | **-** | **-** |
| **PK 001** | **76** | **f** | **34.0** | **-** | **-** | **+** | **+** |
| **PK 002** | **58** | **f** | **33.1** | **-** | **-** | **+** | **-** |
| **PK 003** | **81** | **f** | **34.4** | **-** | **-** | **+** | **-** |
| **PK 004** | **41** | **f** | **39.1** | **-** | **+** | **+** | **+** |
| **PK 005** | **54** | **f** | **36.7** | **-** | **+** | **+** | **-** |
| **PK 006** | **67** | **m** | **39.1** | **-** | **-** | **+** | **-** |
| **PK 007** | **85** | **f** | **31.2** | **-** | **-** | **+** | **+** |
| **PK 008** | **88** | **m** | **27.5** | **-** | **-** | **+** | **+** |
| **PK 009** | **54** | **m** | **36.5** | **-** | **+** | **+** | **+** |
| **PK 011** | **63** | **m** | **31.8** | **-** | **-** | **+** | **+** |
| **PK 012** | **69** | **f** | **27.0** | **+** | **+** | **+** | **+** |
| **PK 014** | **63** | **m** | **25.8** | **-** | **+** | **+** | **+** |
| **PK 016** | **64** | **m** | **39.3** | **-** | **-** | **+** | **+** |
| **PK 017** | **76** | **m** | **27.5** | **-** | **-** | **+** | **+** |
| **PK 019** | **69** | **m** | **26.1** | **-** | **-** | **+** | **+** |
| **PK 020** | **71** | **m** | **25.9** | **+** | **+** | **+** | **-** |
| **PK 022** | **77** | **f** | **23.4** | **-** | **+** | **-** | **-** |
| **PK 023** | **72** | **m** | **24.6** | **-** | **+** | **-** | **-** |
| **PK 025** | **67** | **f** | **27.6** | **-** | **+** | **-** | **-** |
| **PK 027** | **78** | **f** | **30.1** | **-** | **+** | **-** | **-** |
| **RK 001** | **60** | **f** | **54.9** | **+** | **-** | **+** | **+** |
| **RK 002** | **65** | **f** | **34.2** | **-** | **-** | **+** | **+** |
| **RK 003** | **71** | **m** | **32.0** | **+** | **-** | **+** | **+** |
| **RK 004** | **90** | **f** | **25.0** | **-** | **-** | **+** | **+** |
| **RK 005** | **65** | **f** | **27.5** | **-** | **-** | **+** | **+** |
| **RK 006** | **83** | **f** | **21.3** | **-** | **-** | **+** | **+** |
| **RK 007** | **57** | **f** | **32.9** | **-** | **-** | **+** | **+** |
| **RK 008** | **64** | **m** | **31.7** | **+** | **-** | **+** | **-** |
| **RK 009** | **73** | **m** | **39.6** | **+** | **+** | **+** | **-** |
| **RK 010** | **61** | **f** | **28.1** | **-** | **-** | **+** | **+** |
| **RK 011** | **71** | **m** | **35.4** | **-** | **-** | **+** | **+** |
| **RK 012** | **55** | **m** | **23.6** | **-** | **-** | **+** | **+** |
| **RK 013** | **69** | **f** | **33.8** | **-** | **-** | **+** | **+** |
| **RK 014** | **78** | **f** | **26.4** | **+** | **+** | **+** | **+** |
| **RK 015** | **69** | **m** | **29.8** | **+** | **+** | **+** | **+** |
| **RK 016** | **88** | **f** | **28.3** | **-** | **-** | **+** | **-** |
| **RK 017** | **46** | **f** | **32.8** | **+** | **-** | **+** | **+** |
| **RK 018** | **69** | **f** | **33.7** | **-** | **-** | **+** | **+** |
| **RK 020** | **78** | **m** | **29.4** | **-** | **-** | **+** | **+** |
| **RK 021** | **41** | **m** | **28.3** | **-** | **+** | **+** | **+** |
| **RK 023** | **57** | **f** | **43.0** | **-** | **+** | **+** | **+** |
| **RK 024** | **72** | **m** | **36.4** | **-** | **-** | **+** | **+** |
| **RK 026** | **75** | **m** | **31.0** | **-** | **+** | **+** | **+** |
| **RK 219 AF** | **67** | **f** | **31.6** | **+** | **-** | **-** | **-** |
| **RK 225 AF** | **60** | **f** | **32.8** | **+** | **-** | **-** | **-** |
| **RK 228 AF** | **61** | **m** | **28.1** | **+** | **-** | **-** | **-** |
| **RK 241 AF** | **60** | **f** | **35.4** | **+** | **-** | **-** | **-** |
| **RK 243 AF** | **80** | **m** | **23.8** | **+** | **-** | **-** | **-** |

P = primary surgery, R = revision surgery, H = hip, K = knee, AF = arthrofibrosis

The BMI of the cohort with primary hip implantations was 28.0±3.9 (range 22.1-34.9) for 23 patients (7 male, 16 female). The BMI of the cohort with primary knee implantations was 31.0±5.2 (range 23.4-39.3) for 20 patients (10 male, 10 female). The BMI of the cohort with revision hip implantations was 27.2±6.8 (20.0-51.9) for 25 patients (13 male, 12 female). The BMI of the cohort with revision knee implantations was 31.8±6.6 (21.3-54.9) for 28 patients (12 male, 16 female).
